# Overcoming Energetic Barriers in Acetogenic C1 Conversion

**DOI:** 10.3389/fbioe.2020.621166

**Published:** 2020-12-23

**Authors:** Alexander Katsyv, Volker Müller

**Affiliations:** Department of Molecular Microbiology & Bioenergetics, Institute of Molecular Biosciences, Johann Wolfgang Goethe University, Frankfurt am Main, Germany

**Keywords:** carbon capture, biofuels, electron transport, biohydrogen, hydrogen storage

## Abstract

Currently one of the biggest challenges for society is to combat global warming. A solution to this global threat is the implementation of a CO_2_-based bioeconomy and a H_2_-based bioenergy economy. Anaerobic lithotrophic bacteria such as the acetogenic bacteria are key players in the global carbon and H_2_ cycle and thus prime candidates as driving forces in a H_2_- and CO_2_-bioeconomy. Naturally, they convert two molecules of CO_2_ *via* the Wood-Ljungdahl pathway (WLP) to one molecule of acetyl-CoA which can be converted to different C2-products (acetate or ethanol) or elongated to C4 (butyrate) or C5-products (caproate). Since there is no net ATP generation from acetate formation, an electron-transport phosphorylation (ETP) module is hooked up to the WLP. ETP provides the cell with additional ATP, but the ATP gain is very low, only a fraction of an ATP per mol of acetate. Since acetogens live at the thermodynamic edge of life, metabolic engineering to obtain high-value products is currently limited by the low energy status of the cells that allows for the production of only a few compounds with rather low specificity. To set the stage for acetogens as production platforms for a wide range of bioproducts from CO_2_, the energetic barriers have to be overcome. This review summarizes the pathway, the energetics of the pathway and describes ways to overcome energetic barriers in acetogenic C1 conversion.

## Introduction

In times of global warming there is an immediate need to reduce green house gas emissions. CO_2_ is by far the most important based on atmospheric concentrations and there are several chemical as well as biological approaches to reduce the atmospheric CO_2_ concentration (Claassen et al., 1999; Dey, 2007; Anwar et al., 2018). One is to reduce CO_2_ emissions at the first place followed by efficient capture and storage of CO_2_ (Benson and Orr, 2008; Leung et al., 2014). Lithotrophic microbes that make their biomass from CO_2_ are prime candidates to solve both problems (Götz et al., 2016; Liew et al., 2016; Dürre, 2017; Heijstra et al., 2017). Traditionally, biotechnological processes that compete with oil-based processes use sugars to produce high-value end products (Naik et al., 2010). These approaches do not only produce CO_2_ but also compete for sugar that is a feedstock for humans (Ajanovic, 2011). In contrast, lithotrophic organisms do not produce but use CO_2_ as feedstock (Bengelsdorf et al., 2018). Lithotrophs fix CO_2_ for biomass production with energy derived from solar energy (photolithoautotrophs) or from the oxidation of an inorganic electron donor such as H_2_ (chemolithoautotrophs). The latter occurs in the dark and the absence of oxygen, and thus operates at low costs (Rittmann and Herwig, 2012). Lithotrophic, H_2_-oxidizing, CO_2_ fixing microorganisms are the methanogenic archaea that only produce methane (Wolfe, 1971; Zehnder and Brock, 1979; Enzmann et al., 2018) or the acetogenic bacteria that produce different end products such as acetate and ethanol or butyrate and formate (Müller et al., 2004; Drake et al., 2008). They are also metabolically flexible and grow lithotrophically on H_2_ + CO_2_ or on CO, but also heterotrophically on sugars, alcohols, carbonic acids, primary and secondary alcohols (Andreesen et al., 1973; Bache and Pfennig, 1981; Eichler and Schink, 1984; Drake et al., 1997, 2008; Ragsdale and Pierce, 2008; Schuchmann and Müller, 2016). Heterotrophic growth in almost every case goes along with reduction of CO_2_ to acetate (Müller, 2003). Thus, acetogens can couple oxidation of various organic and inorganic electron donors to the reduction of CO_2_ to acetate or the other before mentioned products. Therefore, acetogens are the most flexible organisms to be used for a biological approach to capture and store CO_2_ in the dark and absence of O_2_ (Daniell et al., 2012; Liew et al., 2016; Köpke and Simpson, 2020). Since CO_2_ fixation can be driven by H_2_ oxidation, these bacteria also capture and store H_2_, a key process in the biohydrogen economy (Bailera et al., 2017; Müller, 2019; Schwarz and Müller, 2020).

## Acetogenic Bacteria and the Wood-Ljungdahl Pathway

**Figure 1:**
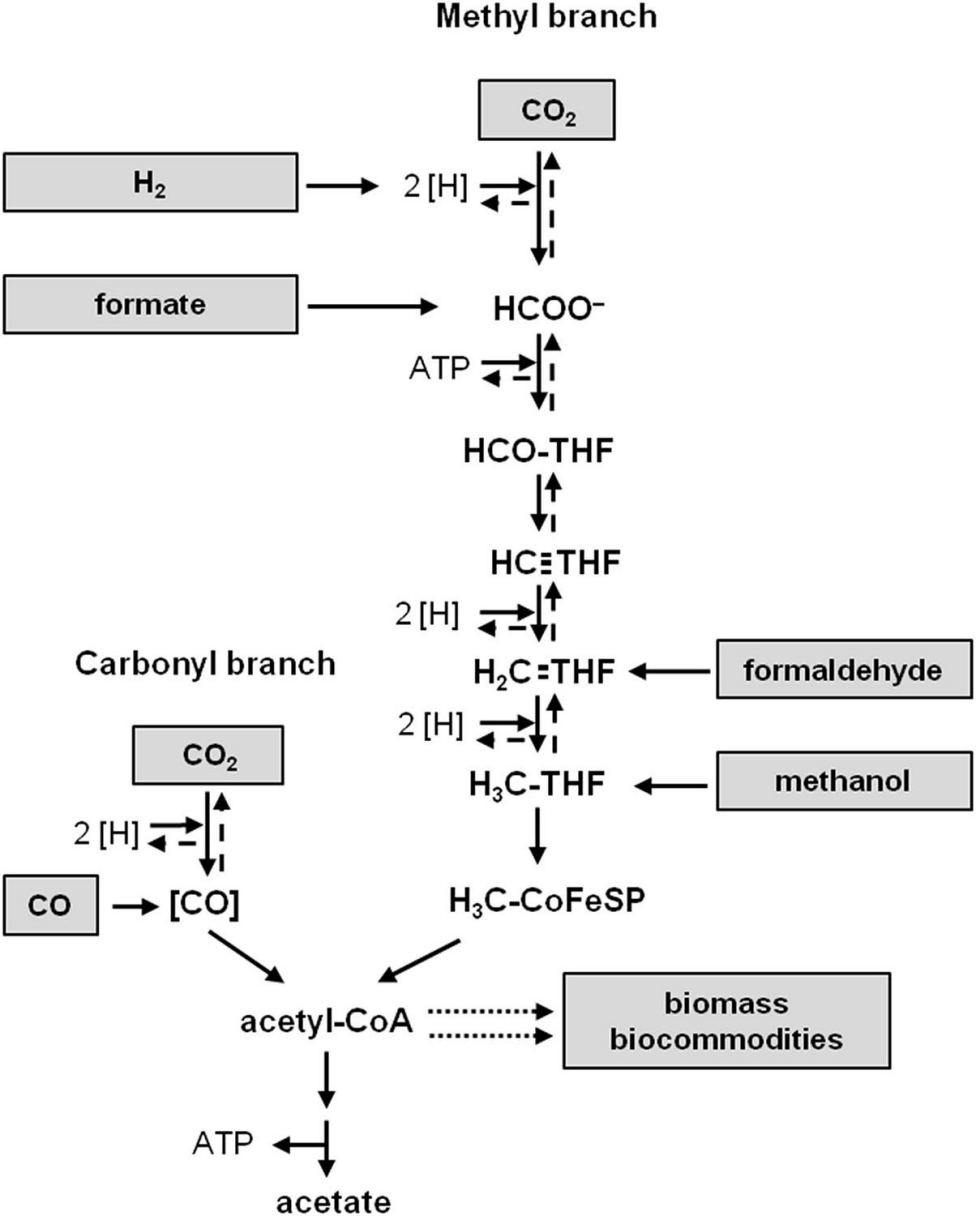
The Wood-Ljungdahl pathway of CO_2_ reduction. Substrates fed directly into the pathway are shown to the left and right. Acetyl-CoA is the precursor of biomass and biocommodities (dotted arrows). [H], reducing equivalents; THF, tetrahydrofolic acid; [CO], enzyme-bound CO; large dashed arrows, oxidative direction of the pathway.

Acetogenic bacteria are a phylogenetically very diverse group of strictly anaerobic bacteria ubiquitous in nature. They are characterized by a reductive pathway in which two mol of CO_2_ are reduced to one mol of acetyl-CoA and then further to acetate, the Wood-Ljungdahl pathway (WLP) (Figure 1; Ljungdahl, 1986; Wood et al., 1986). Reducing power can be derived from the oxidation of organic but also inorganic carbon sources. Some acetogens naturally produce ethanol and butyrate in addition and, therefore, they have come into focus of an alternative, CO_2_-based bioeconomy (Schiel-Bengelsdorf and Dürre, 2012; Dürre, 2017). Ethanol is also produced from CO_2_, H_2_ and CO (syngas) on a large industrial scale (Daniell et al., 2012; Bengelsdorf et al., 2018). Apart from these gaseous substrates used, other C1 substrates such as formic acid or methanol are promising feedstocks for an alternative bioeconomy using acetogens as biocatalysts (Cotton et al., 2019; Müller, 2019). In addition to the mere use as production platforms, acetogens are also promising candidates in the H_2_-economy as potential catalysts for H_2_ storage or production (Bailera et al., 2017; Schwarz et al., 2018; Schwarz and Müller, 2020).

Among the naturally occurring carbon fixation pathways the WLP is the only pathway that does not need additional ATP to operate (Ragsdale and Pierce, 2008; Fuchs, 2011). One mol of CO_2_ is reduced to formate and then bound to the C1-carrier tetrahydrofolate (THF), driven by the hydrolysis of ATP (Himes and Harmony, 1973; Lovell et al., 1988). From the formyl-THF, water is split off and the produced methenyl-THF is further reduced *via* methylene- to methyl-THF (Clark and Ljungdahl, 1982). The latter condenses with CO and coenzyme A (CoA) on the enzyme acetyl-CoA synthase/CO dehydrogenase to acetyl-CoA; the CO derives from the reduction of another CO_2_ by the CO dehydrogenase (Drake et al., 1980; Pezacka and Wood, 1984; Ragsdale and Wood, 1985; Raybuck et al., 1988; Seravalli et al., 1997). Acetyl-CoA is then converted *via* acetyl phosphate to acetate and ATP. Thus the net synthesis of ATP by substrate level phosphorylation is zero. Since the bacteria grow by production of acetate from H_2_ + CO_2_ there must be additional mechanisms to generate ATP (Schaupp and Ljungdahl, 1974; Drake et al., 1981; Müller, 2003).

## Chemiosmotic Energy Conservation in Acetogens

**Figure 2:**
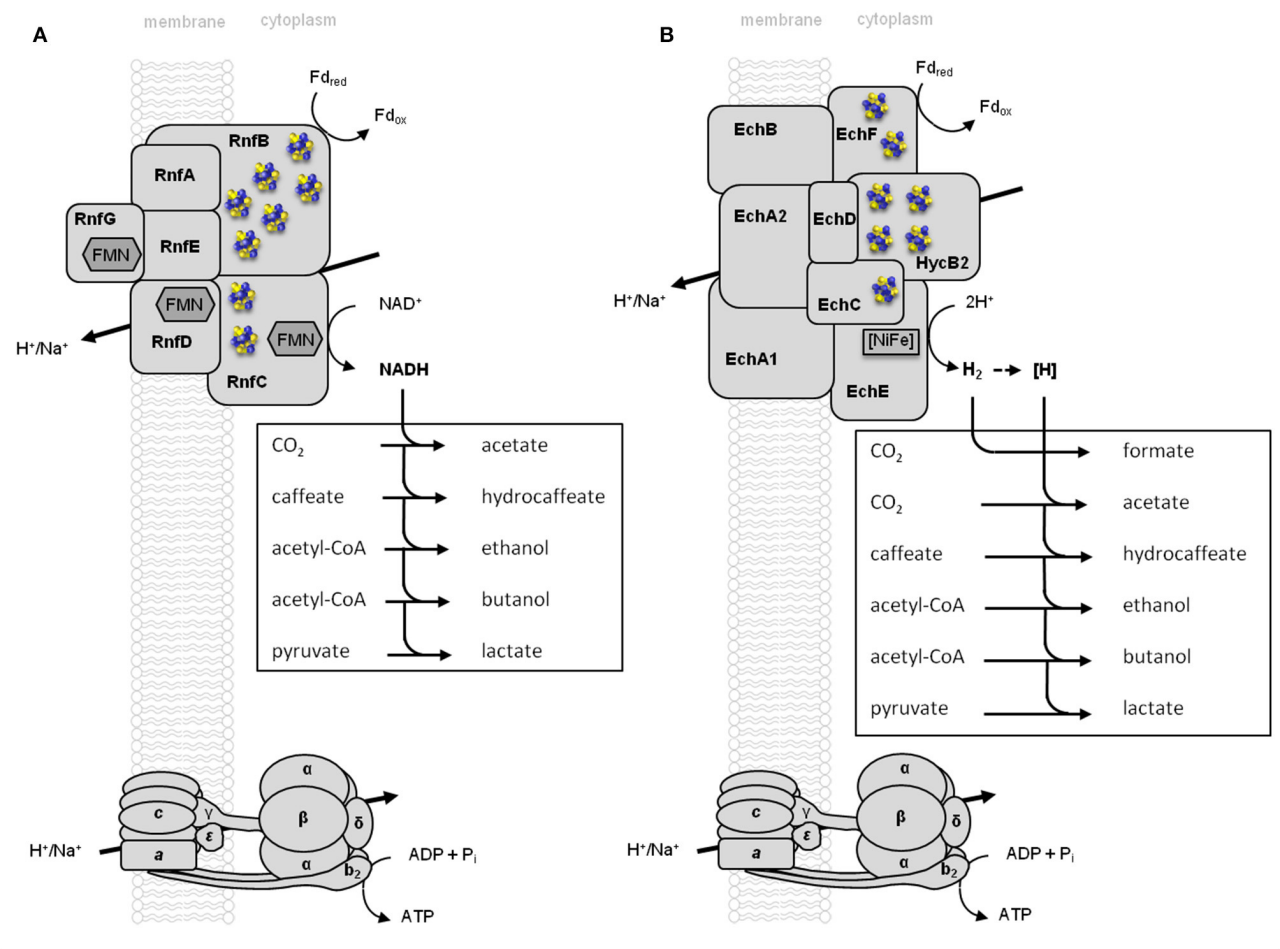
Respiratory enzyme complexes Rnf and Ech in acetogens. Acetogens are classified in Rnf- **(A)** or Ech-containing **(B)** organisms. Exergonic electron transfer leads to the translocation of H^+^/Na^+^ across the cytoplasmic membrane and the electrochemical H^+^/Na^+^ potential is then the driving force for ATP synthesis. Reducing equivalents are oxidized *via* different pathways, results in different product formations. Rnf, model of *A. woodii*; Ech, model of *T. kivui*; [H], reducing equivalents; FMN, flavin mononucleotide.

Currently, acetogens can be divided into two bioenergetic groups, the Rnf- and the Ech-acetogens (Figure 2; Schuchmann and Müller, 2014). The Rnf- and the Ech-complex are membrane-bound respiratory enzymes that both use reduced ferredoxin as reductant (Schmehl et al., 1993; Hedderich and Forzi, 2005; Buckel and Thauer, 2018b). The Rnf complex has six subunits and catalyzes ferredoxin:NAD^+^ oxidoreductase activity; the free energy change of the electron transport is coupled to the extrusion of ions from the cytoplasm to the periplasm thus generating an electrochemical ion gradient across the membrane that drives ATP synthesis *via* a F_1_F_O_ ATP synthase (Müller et al., 2008; Biegel and Müller, 2010; Westphal et al., 2018). The coupling ion maybe Na^+^ as in the case of *Acetobacterium woodii* or H^+^ as suggested for *Clostiridum ljungdahlii* or *Clostridium autoethanogenum* (Biegel and Müller, 2010; Tremblay et al., 2012; Kuhns et al., 2020). Two points are important to make: first, the nature of the coupling ion used has dramatic consequences for the energetic status, especially under stress conditions and second, NADH is the end product of this respiration and can be regarded as a waste product that needs to be recycled (reoxidized). Thus, by metabolic engineering, any reductive, NADH consuming pathway can be hooked up to the Rnf complex. In the second group, the Ech-acetogens, with *Thermoanaerobacter kivui* and *Moorella thermoacetica* as model strains, reduced ferredoxin is oxidized by an 8- or 9-subunit, membrane-bound enzyme that transfers the electron to protons, thus producing molecular H_2_ (Pierce et al., 2008; Schoelmerich and Müller, 2020). This electron transport is coupled to the export of ions across the cytoplasmic membrane and the electrochemical ion gradient established drives the synthesis of ATP *via* a F_1_F_O_ ATP synthase (Welte et al., 2010; Schoelmerich and Müller, 2019). Three points are important to make here: first, the nature of the ferredoxin involved and its redox potential is not known. *A. woodii*, for example, has seven genes potentially encoding a ferredoxin. Since the CO_2_/CO couple (E_0_' [CO_2_/CO] = −520 mV) requires a low potential reductant we calculate with a redox potential of −450 to −500 mV. Second, the potential difference between reduced ferredoxin (E_0_' [Fd^2−^/Fd] ~ −450 to −500 mV) and protons (E_0_' [H_2_/H^+^] = −414 mV) is only about half (ΔE_0_' = +86 mV to +36 mV) of the difference of the redox couple reduced ferredoxin:NAD^+^ (ΔE_0_' = +180 mV) and thus, only about half the amount of ions can be translocated per electron transported. These numbers have not been determined for any Rnf- or Ech-complex but based on thermodynamics we consider two ions per two electrons in Rnf and one ion per two electrons in Ech as maximum. Third, the product of respiration is the volatile gas H_2_ and thus there is no need for a reductive pathway to be hooked up to the respiration since the electron escapes into the environment as gas. Although this is attractive for the cells it can be deleterious to the cell if the H_2_ partial pressure in the ecosystem is so high that it exhibits a thermodynamic backup pressure on the enzyme so that respiration comes to an end. The ΔG^0′^ of the reaction ferredoxin:H^+^ is only −7 to −16.6 kJ/mol. This allows H_2_ production only up to a partial pressure of 0.05 MPa.

The function of Rnf as respiratory enzyme has been proven by genetic (Westphal et al., 2018) and biochemical experiments (Biegel and Müller, 2010) and recently, the final proof has been obtained using the enzyme from a thermophilic fermenting bacterium, *Thermotoga maritima* (not an acetogen) (Kuhns et al., 2020) as well as the acetogen *A. woodii* (Wiechmann and Müller, unpublished data). The enzyme was purified and reconstituted into liposomes and catalyzed electron transfer from reduced ferredoxin to NAD^+^; this electron transport was coupled to a primary and electrogenic Na^+^ transport into the lumen of the liposomes (Wiechmann and Müller, unpublished data). For Ech, there is evidence that inverted membrane vesicles of the acetogen *T. kivui* couple H^+^ as well as Na^+^ transport into the lumen of the vesicles to H_2_ production from reduced ferredoxin (Schoelmerich and Müller, 2019). However, this organism has two Ech-encoding gene clusters and the final proof of ion translocation and the ion used has to await genetic studies and its purification and reconstitution into liposomes. Ion transport coupled to Ech-catalyzed reaction has also been observed in vesicles of the methanogenic archaeon *Methanosarcina mazei* (Welte et al., 2010) and there is no reason to believe that Ech is not as respiratory enzyme in acetogens. This is different in cytochrome-containing acetogens. *A. woodii* and *T. kivui* do not contain cytochromes, but some acetogens like *M. thermoacetica* do. Historically, the discovery of cytochromes in the acetogens *Clostridium formicoaceticum* and *M. thermoacetica* was an exciting discovery since it immediately argued for a respiratory chain involved in acetogenesis (Gottwald et al., 1975). Unfortunately, 45 years after their discovery there is no evidence for that. Indeed, it may well be that cytochromes are not involved in acetogenesis (CO_2_ reduction) but reduction of alternative electron acceptors such as nitrate (Visser et al., 2016). Since the involvement of cytochromes in acetogenesis is still a molecular hallucination, we will not discuss this further in this review.

The ion gradient established by the Rnf and Ech complex is then used by a F_1_F_O_-ATP-synthase to drive the synthesis of ATP. F_1_F_O_-ATP-synthases have been purified from only a few acetogens such as *M. thermoacetica* (Ivey and Ljungdahl, 1986), *Moorella thermoauthotrophica* (Das et al., 1997), *A. woodii* (Reidlinger and Müller, 1994) and *Eubacterium limosum* (Litty and Müller, 2020). F_1_F_O_-ATP-synthases are macromolecular machines that convert electrochemical energy via mechanical energy into chemical energy (ATP) (Müller and Grüber, 2003). They are composed of two motors that are connected by a central stalk. Ion flow through the membrane-embedded motor made of the rotor (a ring of *c* subunits) and the stator (*a* subunit) drives rotation of the rotor against the stator. Furthermore, rotation of the *c*-ring drives rotation of the central stalk that interacts with the three ATP synthesizing centers in the hydrophilic F_1_ domain, leads to the synthesis of ATP from ADP + P_i_. The *c* subunit harbors the ion binding site which are protons in most ATP synthases (Müller and Grüber, 2003; Meier et al., 2006; Grüber et al., 2014) and Na^+^ in a few (Meier et al., 2009; Brandt and Müller, 2015; Mayer et al., 2015). Protons are bound to the so-called active carboxylate in helix two of the *c* subunit (a aspartate or glutamate residue) (Fillingame et al., 2003) whereas the conserved Na^+^-binding site has in addition two residues, a glutamine in helix one and a serine or threonine residue downstream of the active carboxylate (Rahlfs and Müller, 1999; Müller and Grüber, 2003). There are more residues involved in complexing Na^+^ (Meier et al., 2005) but these three are the conserved ones that make the conserved Na^+^-binding motif. *C. ljungdahlii* and *C. autoethanogenum* only have the active carboxylate but not the Na^+^-binding motif (Mayer et al., 1986), whereas *A. woodii* (Reidlinger et al., 1994; Matthies et al., 2014) as well as *E. limosum* (Litty and Müller, 2020) have the conserved Na^+^-binding motif. The activity of the purified enzymes is strictly Na^+^ dependent and the enzymes translocate Na^+^ after reconstitution into liposomes (Fritz and Müller, 2007; Litty and Müller, 2020).

A critical number for bioenergetic calculations is the number of *c* subunits per ring because this gives the number of ions translocated for the synthesis of three moles of ATP. This number has been experimentally determined only for *A. woodii*; in this case the *c* ring has 10 Na^+^-binding sites (Matthies et al., 2014). Divided by three ATP synthesizing centers that gives 3.3 Na^+^/ATP. The non-acetogenic *Clostridium paradoxum* has 11 H^+^-binding sites, giving a stoichiometry of 3.6 H^+^/ATP (Ferguson et al., 2006; Meier et al., 2006). This number is used in this review to calculate the overall ATP yields in *C. autoethanogenum*.

## Electron Carriers in the WLP

After having discussed that acetogens have either one of the two respiratory enzymes Rnf or Ech the obvious question is: what are the cofactors of the other dehydrogenases/reductases in the WLP and how is the pool of reduced ferredoxin maintained. The first enzyme in the carbonyl branch is the acetyl-CoA synthase/CO dehydrogenase which is also the key enzyme in the entire pathway (Drake et al., 1980; Pezacka and Wood, 1984; Ragsdale and Wood, 1985; Raybuck et al., 1988; Seravalli et al., 1997). Due to the low redox potential of the CO_2_/CO couple (E_0_' [CO_2_/CO] = −520 mV) only reduced ferredoxin can act as reductant here. How ferredoxin (E_0_' [Fd^2−^/Fd] ~ −450 to −500 mV) is reduced with H_2_ (E_0_' [H_2_/H^+^] = −414 mV) has been an enigma for a long time. With the discovery of electron bifurcation to couple endergonic with exergonic redox reactions (Li et al., 2008; Buckel and Thauer, 2018a), the solution was at hand: an electron-bifurcating hydrogenase (Schut and Adams, 2009). Such an enzyme is indeed also present in *A. woodii*. It couples the exergonic electron flow from H_2_ to NAD^+^ (E_0_' [NAD^+^/NADH] = −320 mV) to the endergonic electron flow from H_2_ to ferredoxin (Schuchmann and Müller, 2012). In this context it is important to note that electron bifurcation is often mistakenly considered as mechanism for energy conservation. It is not, but it saves cellular energy. If electron transport from H_2_ to ferredoxin would be driven by ATP, at least one ATP would have to be invested. With electron bifurcation the equivalent of only a fraction of an ATP can be invested. This kind of energy saving is a prerequisite for life at the thermodynamic limit as in acetogens. Therefore, acetogens employ a multitude of different electron-bifurcating enzymes in their metabolism and, actually most of the various electron-bifurcating enzymes known to date are from acetogens (Müller et al., 2018).

The first step in the methyl branch is the reduction of CO_2_ to formate. Since the redox potential of the CO_2_/formate couple is −420 mV, neither NAD^+^ nor NADP^+^ can be used as reductant. The solution is different in different acetogens: some use a ferredoxin-dependent formate dehydrogenase, others a combination of an electron-bifurcating hydrogenase and an electron-bifurcating formate dehydrogenase and still others use a H_2_-dependent CO_2_ reductase (HDCR) (Yamamoto et al., 1983; Nagarajan et al., 2013; Schuchmann and Müller, 2013; Wang et al., 2013a,b). In the latter, H_2_ is oxidized by a hydrogenase subunit of the HDCR and the electrons are transferred, most likely by two small iron-sulfur containing electron transfer subunits, to the formate dehydrogenase (Schuchmann and Müller, 2013). This is energetically feasible given the redox potential E_0^′^_ of −420 mV of the redox couple CO_2_/formate and −414 mV of the H_2_/2 H^+^ couple. In the electron-bifurcating formate dehydrogenase of *C. autoethanogenum*, the same is achieved but by two consecutive reactions: first, a NADP^+^-specific electron-bifurcating hydrogenase reduces NADP^+^ and ferredoxin and second, an electron-bifurcating formate dehydrogenase uses NADP^+^ and reduced ferredoxin for CO_2_ reduction (Wang et al., 2013a). The next reduction step is the reduction of methenyl-THF to methylene-THF, catalyzed by the methylene-THF dehydrogenase. This enzyme maybe NAD^+^ specific as in *A. woodii* or NADP^+^ specific as in *C. autoethanogenum* or *Sporomusa ovata* (O'Brien et al., 1973; Ragsdale and Ljungdahl, 1984; Wang et al., 2013a; Kremp et al., 2020). The last reduction step is the reduction of methylene-THF to methyl-THF, catalyzed by the methylene-THF reductase (MTHFR). This enzyme is of special interest in the bioenergetics of acetogens and it was proposed 43 years ago by Thauer et al. (1977) to be an energetic coupling site. This is based on the high redox potential of the pair methylene-/methyl-THF of −200 mV, arguing that its reduction with NADH would deliver sufficient energy to be used in energy conservation (Wohlfarth and Diekert, 1991). Accordingly, it was speculated for a long time that the methylene-THF reductase is the terminal acceptor of a respiratory chain that energizes the cytoplasmic membrane for ATP synthesis (Müller, 2003). However, with the genomic sequences for acetogens and the biochemical data available this can be ruled out for the model strains analyzed. Nevertheless, the enzyme could use electron bifurcation (Buckel and Thauer, 2018a; Müller et al., 2018) to couple the exergonic reduction of methylene- to methyl-THF with the endergonic reduction of ferredoxin which then drives energy conservation in the respiratory chain. Indeed, the enzymes from *M. thermoacetica* or *C. ljungdahlii* do not couple NADH oxidation to methylene-THF reduction, indicating that a second electron acceptor is missing (Moore et al., 1974; Clark and Ljungdahl, 1984; Mock et al., 2014). Since it is a characteristic of electron-bifurcating enzymes that they are only active in presence of all three reaction partners, the lack of activity was taken to suggest that the enzyme is electron bifurcating to an unknown second acceptor (Mock et al., 2014). However, this still needs to be verified. In contrast, *A. woodii* has a NAD^+^-dependent, non-electron-bifurcating methylene-THF reductase (Bertsch et al., 2015). In sum, the electron carriers used by the enzymes of the WLP are very different.

## Electron Carriers Involved in Substrate Oxidation

The diversity also holds true for the electron carriers involved in substrate oxidation. Sugars such as glucose, fructose, mannitol are oxidized by the Embden-Meyerhof-Parnas pathway with NAD^+^ in the glycerol-3-phosphate dehydrogenase reaction and ferredoxin in the pyruvate:ferredoxin oxidoreductase reaction as electron carriers. Other substrates such as ethanol only yield NADH and still others such as formate only yield molecular H_2_ (Schuchmann and Müller, 2013; Bertsch et al., 2016). During lithotrophic growth, H_2_ is oxidized by an electron-bifurcating hydrogenase yielding both, NADH and reduced ferredoxin (Schuchmann and Müller, 2012). In Ech-containing acetogens, H_2_ oxidation could theoretically yield reduced ferredoxin by Ni-Fe hydrogenases (Ech-complex), as shown in the methanogenic archaeon *Methanosarcina barkeri* (Meuer et al., 2002). CO is oxidized by the CO dehydrogenase coupled to reduction of ferredoxin (Seravalli et al., 1997). In sum, oxidation of different substrates yields different reduced electron carriers such as NADH, NADPH, reduced ferredoxin, H_2_ or combinations thereof, whereas the WLP only accepts, species specific, distinct electron donors in the right stoichiometry. Thus, there is an essential need to switch and adjust the electron carriers between the oxidation module and the WLP.

## Electron Carrier Adjustment: Many Variations of a Theme

Conversion of redox carriers into each other requires energy and since acetogens life at the thermodynamic edge of life, they have evolved energy-efficient ways. They employ specialized membrane-bound and soluble enzyme systems. The membrane-bound enzymes are the before discussed respiratory enzymes Rnf and Ech. They are both coupled reversibly to the membrane potential. In the above described function they serve to reduce NAD^+^ (E_0_' [NAD^+^/NADH] = −320 mV) with reduced ferredoxin E_0_' [Fd^2−^/Fd] ~ −450 to −500 mV) as reductant or to reduce protons to H_2_ (E_0_' [H_2_/2 H^+^] = −414 mV). During growth on low energy substrates that only yield NADH, *A. woodii* employs the Rnf to drive reverse electron transport (Hess et al., 2013b; Westphal et al., 2018). This is actually the function in most organisms and this function was decisive for the name giving: *R*hodobacter *n*itrogen *f*ixation (Schmehl et al., 1993). There, the Rnf complex drives the endergonic reduction of ferredoxin, required for nitrogen fixation, with NADH as reductant (Schmehl et al., 1993; Kumagai et al., 1997; Saeki and Kumagai, 1998). Analogously, the Ech complex can drive the endergonic reduction of ferredoxin with H_2_ as reductant at the expense of the electrochemical ion gradient across the membrane (Meuer et al., 2002). The same function is achieved by the soluble electron-bifurcating hydrogenase that couples H_2_ oxidation to the reduction of both, NAD^+^ (exergonic) and ferredoxin (endergonic) where the energy is provided by electron bifurcation (Schuchmann and Müller, 2012). Conversion of NADP^+^ and NAD^+^ is catalyzed with reduced ferredoxin as driver in the Nfn and Stn-type electron-bifurcating transhydrogenases found in acetogens and the electron-bifurcating hydrogenase/formate dehydrogenase complex connects cellular H_2_, NADP^+^, ferredoxin and CO_2_ pools (Wang et al., 2010; Nguyen et al., 2017; Kremp et al., 2020).

**Figure 3:**
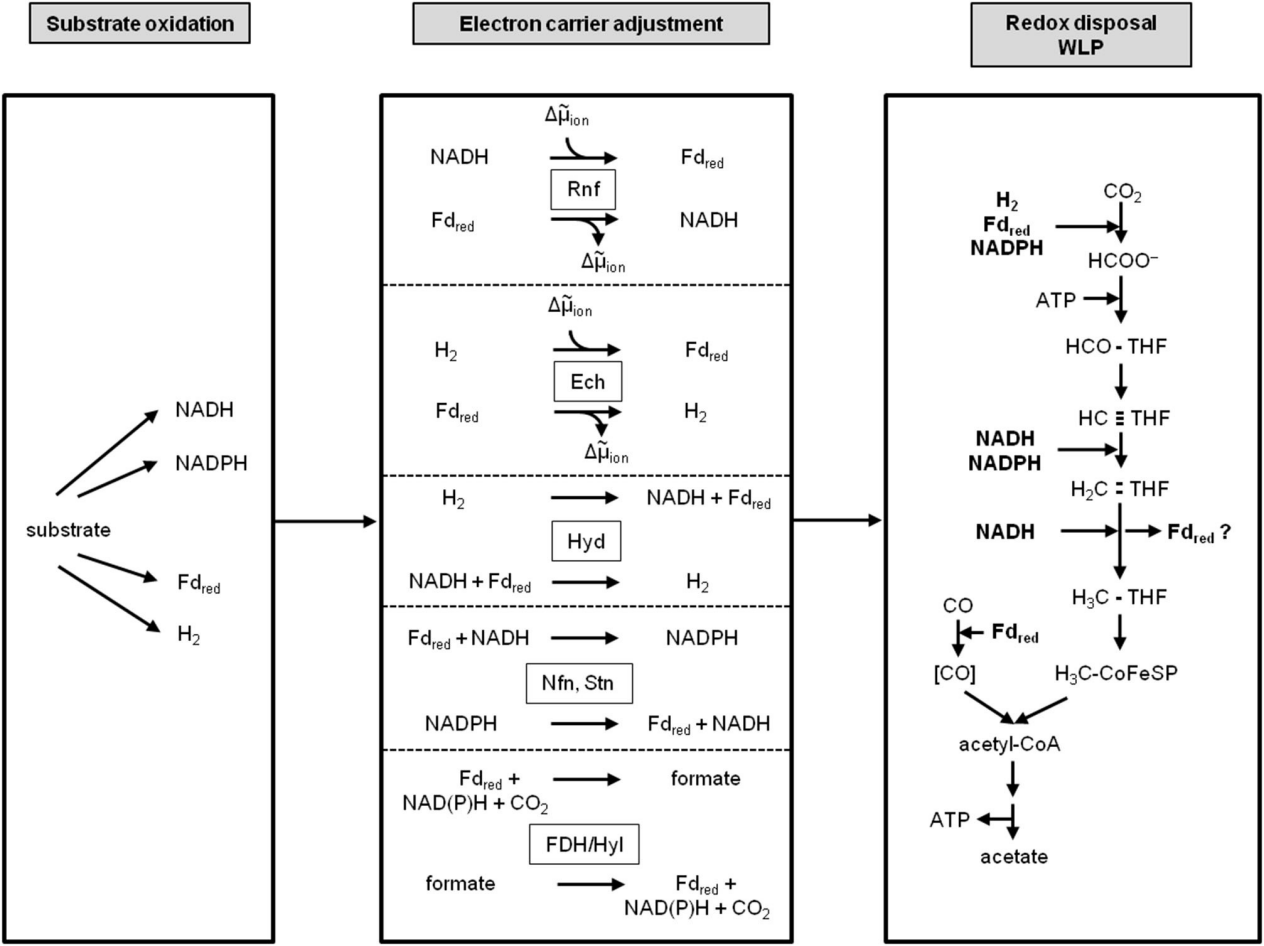
Modularity of acetogenesis. Depending on the redox potential of the substrate, redox carriers with different redox potentials are reduced. The WLP requires species-specific, fixed sets of distinct electron carriers with different redox potentials. To switch electron carriers between the substrate oxidation module and the WLP, a third module that adjusts the electron carriers is required; it contains enzymes like Rnf, Ech, electron-bifurcating hydrogenase, Nfn/Stn or the electron-bifurcating hydrogenase/formate dehydrogenase complex. THF, tetrahydrofolic acid; Hyd, electron-bifurcating hydrogenase; FDH/Hyl, electron-bifurcating formate dehydrogenase; Nfn, electron-bifurcating, ferredoxin-dependent transhydrogenase; Stn, *Sporomusa* type Nfn.

Together with the difference in electron carrier specificity of the substrate oxidation reaction and the WLP and the different respiratory enzymes this gives hundreds of possible combinations for energy conservation (Figure 3). Since acetogens growing on H_2_ + CO_2_ make only a fraction of an ATP per mol of acetate formed, a change in the electron carrier specificity of a given metabolic scheme makes a huge difference for the ATP yield. Therefore, reliable calculation for the overall ATP gain can only be done with clear conscience for those organisms in which the electron carrier specificity of the redox reaction and the type of respiratory enzyme has been determined experimentally. Metabolic schemes with too many unknown variables are rather harmful than useful. Therefore, we will concentrate on the well-studied acetogen *A. woodii* that grows very robust on H_2_ + CO_2_ and is a prime candidate also for a biohydrogen economy, but that does not grow on CO or syngas, and on *C. autoethanogenum* (or its close relative, *C. ljungdahlii*), a working horse in the industrial production of biofuels from syngas but a less well-understood metabolism.

## Bioenergetics of *A. woodii* and *C*. *autoethanogenum*

Acetogenesis from H_2_ + CO_2_ or CO according to equations 1 and 2 goes along with a free energy change of −95 and −175 kJ/mol.

(1) $$\begin{array}{l} 4{\text{ H}}_{2}+2\text{ C}{\text{O}}_{2}\to 1\text{ C}{\text{H}}_{3}\text{CO}{\text{O}}^{-}+1{\text{ H}}^{+}+2{\text{ H}}_{2}\text{O}\\ \;\Delta {\text{G}}^{0^{\text{′}}}=-95\text{ kJ}/\text{mol} \end{array}$$

(2) $$\begin{array}{l} 4\text{ CO}+2{\text{ H}}_{2}\text{O}\to 1\text{ C}{\text{H}}_{3}\text{CO}{\text{O}}^{-}+1{\text{ H}}^{+}+2\text{ C}{\text{O}}_{2}\\ \;\Delta {\text{G}}^{0^{\text{′}}}=-175\text{ kJ}/\text{mol} \end{array}$$

**Figure 4:**
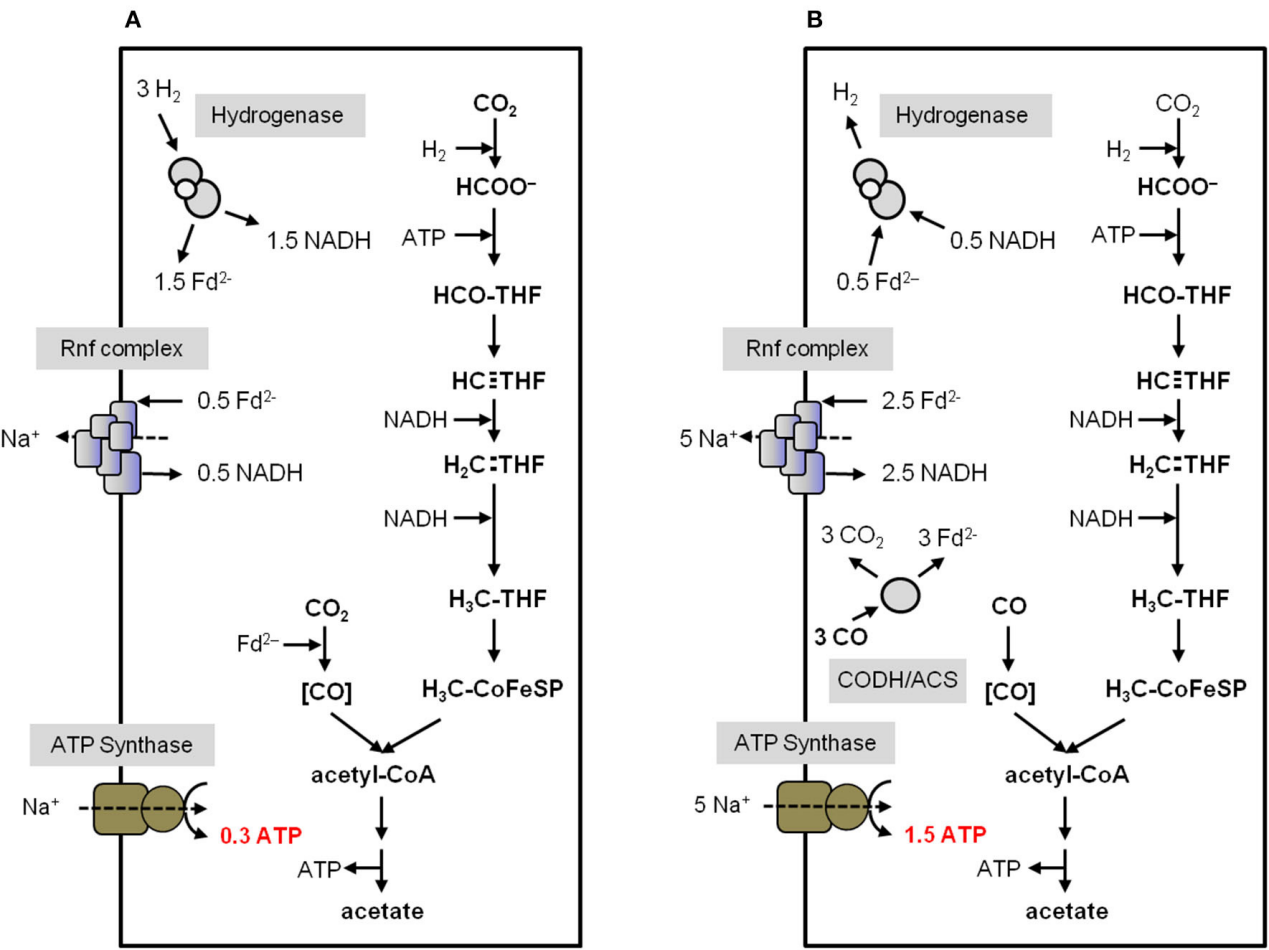
Bioenergetics of acetate formation from H_2_ + CO_2_ and CO in *A. woodii*. The reducing equivalents for the reductive steps in the WLP **(A)** are provided by an H_2_-oxidizing, electron-bifurcating hydrogenase which reduces ferredoxin and NAD^+^. The reducing equivalents for the reductive steps during CO oxidation **(B)** are provided by the CO-oxidizing CODH/ACS which reduces ferredoxin. Excess Fd^2−^ is oxidized by the Rnf complex which reduces NAD^+^ and builds up a Na^+^ gradient. This gradient drives ATP synthesis *via* the Na^+^-dependent ATP synthase. In total, 0.3 ATP from H_2_ + CO_2_ and 1.5 ATP from CO can be synthesized per acetate produced. CODH/ACS, CO dehydrogenase/acetyl coenzyme A synthase; THF, tetrahydrofolic acid.

**Figure 5:**
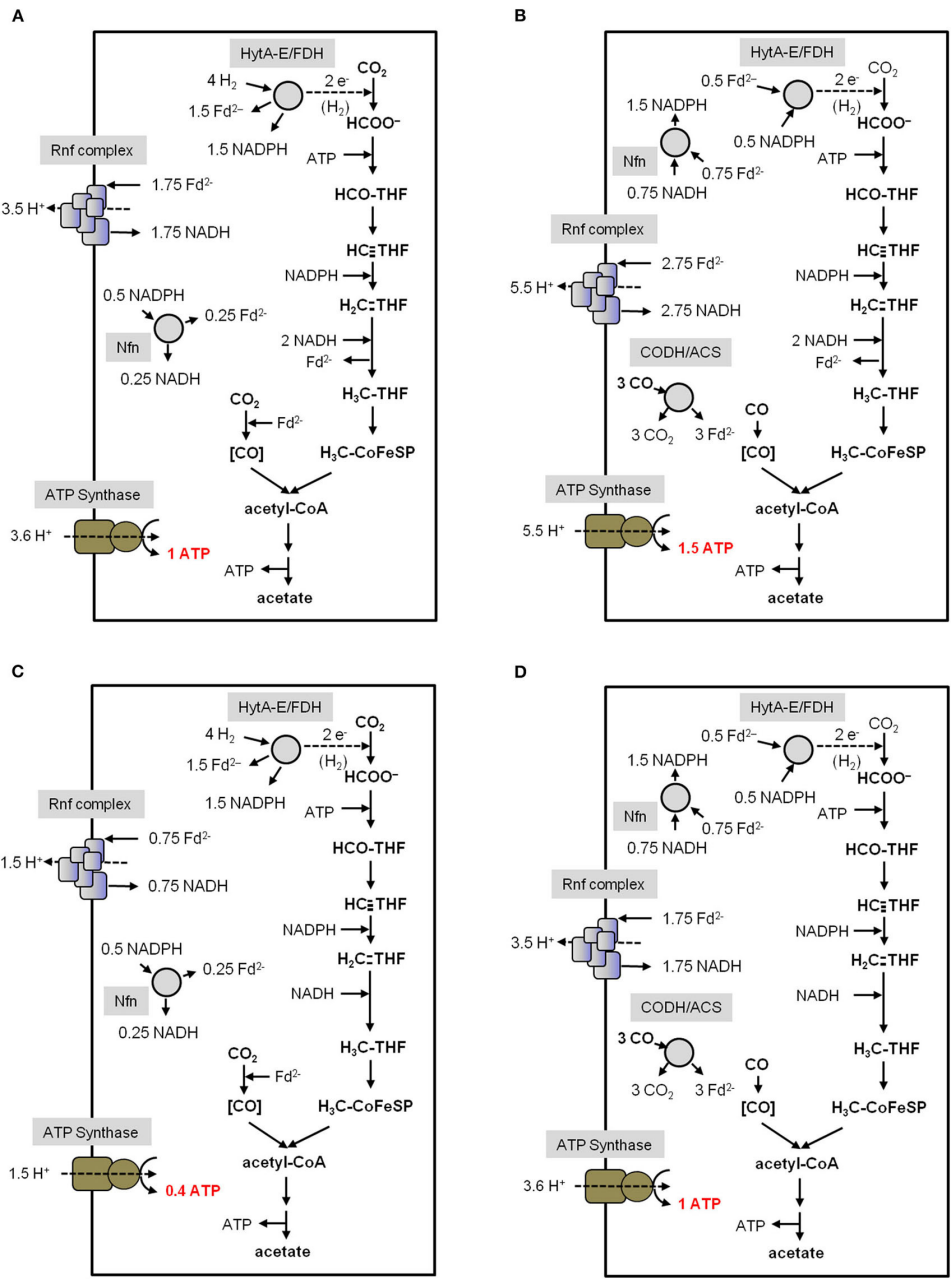
Bioenergetics of acetate formation from H_2_ + CO_2_ and CO in *C. autoethanogenum*. The reducing equivalents for the reductive steps in the WLP **(A,C)** are provided by an H_2_-oxidizing, electron-bifurcating hydrogenase/formate dehydrogenase complex (HytA-E/FDH) which reduces Fd, NADP^+^ and CO_2_. The reducing equivalents for the reductive steps during CO oxidation **(B,D)** are provided by the CO-oxidizing CODH/ACS which reduces Fd. The Nfn complex is transferring electrons between Fd, NADH and NADPH. The methylene-THF reductase is assumed to be electron bifurcating in **(A,B)**. In **(C,D)** the methylene-THF reductase is not electron bifurcating. Excess Fd^2−^ is oxidized by the Rnf complex which reduces NAD^+^ and builds up a H^+^ gradient. This gradient drives ATP synthesis *via* the H^+^-dependent ATP synthase. In total, 0.4/1 ATP from H_2_ + CO_2_ and 1/1.5 ATP from CO (depending on the MTHFR reaction) can be synthesized per acetate produced. CODH/ACS, CO dehydrogenase/acetyl coenzyme A synthase; THF, tetrahydrofolic acid; Nfn, electron-bifurcating and ferredoxin-dependent transhydrogenase.

Considering the H_2_ and CO concentration in the environment this is sufficient for only 0.3 and 1.5 mol ATP/mol acetate, respectively (Kim and Hegeman, 1983; Novelli et al., 1999; Schuchmann and Müller, 2014). The enzymology and bioenergetics of *A. woodii* and *C. autoethanogenum* for acetogenesis from H_2_ + CO_2_ or CO is summarized in Figures 4 and 5. In *A. woodii*, not only the electron carriers are known but also the ion/ATP stoichiometry of the ATP synthase is known (3.3 Na^+^/ATP), the only case for acetogens (Matthies et al., 2014). This allows to calculate the ATP/acetate stoichiometry very accurately with 0.3 mol ATP/mol of acetate with H_2_ + CO_2_ as substrate (Figure 4A). *A. woodii* does not grow on CO (Bertsch and Müller, 2015) but resting cells are able to oxidize CO according to equation 2 (Diekert et al., 1986). This yields 1.5 ATP/mol of acetate (Figure 4B).

**Figure 6:**
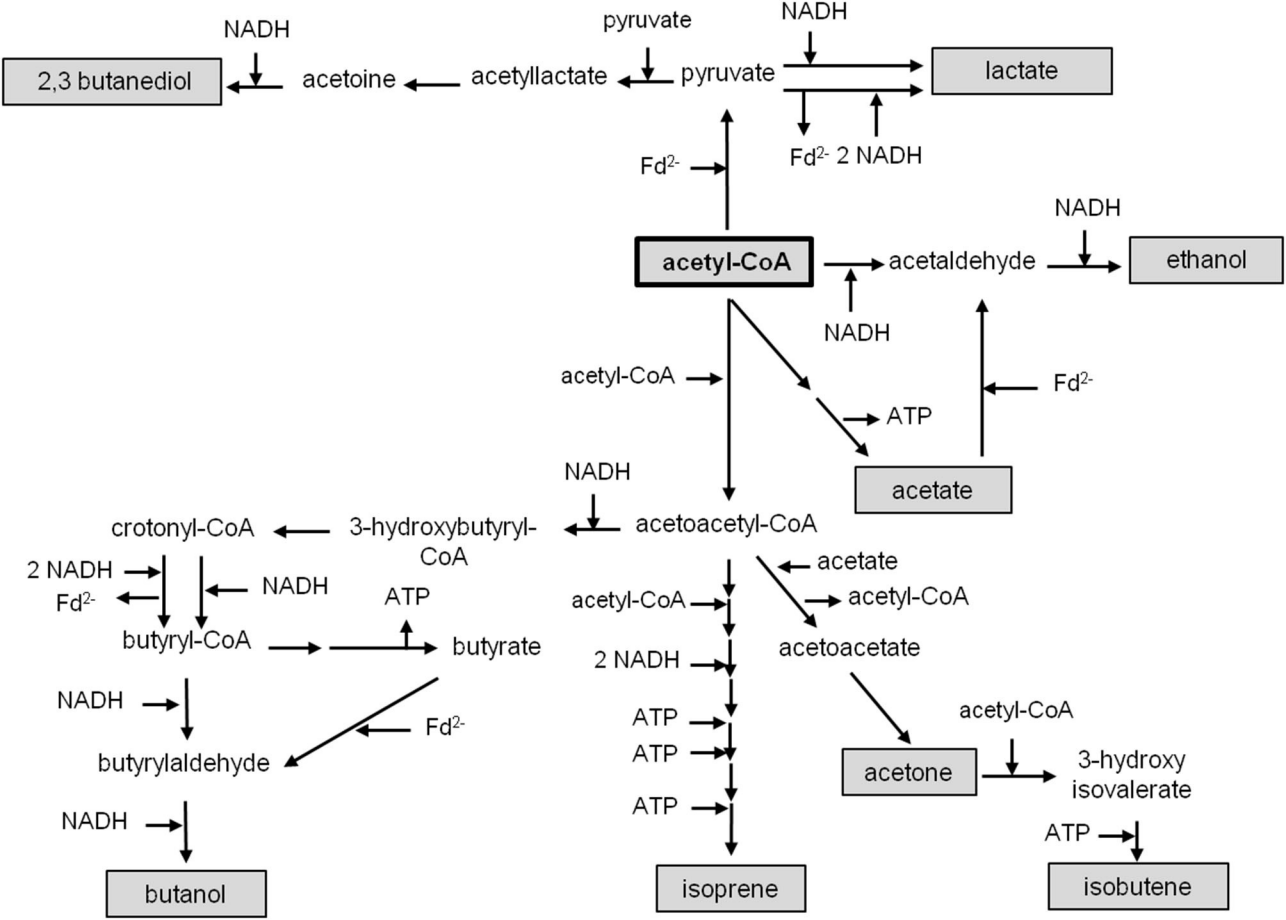
Metabolic pathways of acetyl-CoA reduction. Acetyl-CoA is synthesized *via* the Wood–Ljungdahl pathway and can be converted to lactate, 2,3 butanediol, ethanol, acetate, butanol, isoprene, acetone or isobutene. Cofactors involved in different metabolic pathways are indicated.

For *C. autoethanogenum*, there are two unknowns: the ion/ATP stoichiometry and whether or not the methylene-THF reductase (MTHFR) is electron bifurcating. For the calculations we have assumed an ion/ATP stoichiometry of 3.6 (see above) and for the methylene-THF reductase we assume either electron bifurcation with ferredoxin as second electron acceptor or no electron bifurcation. With these two variables, the ATP gain in *C. autoethanogenum* varies from 0.4 to 1 mol/mol of acetate with H_2_ + CO_2_ as substrate (Figures 5A,C). It should be mentioned that the ATP yield of acetogenesis from H_2_ + CO_2_ in *C. autoethanogenum* is higher compared to *A. woodii* if an electron-bifurcating methylene-THF reductase is assumed (Figures 5A,B). With CO as electron donor, the energetic of acetogenesis is much better. According to equation 2, the ΔG^0′^ is −175 kJ/mol and acetate formation from CO in *C. autoethanogenum* goes along with the synthesis of 1–1.5 mol ATP/mol acetate (Figures 5B,D). This is more than from H_2_ + CO_2_ but both values are still very low compared to fermenting bacteria or even aerobes (Müller, 2008). However, the situation gets worse when products other than acetate are formed from acetyl-CoA (Figure 6).

## Bioenergetics of the Formation Of Products Other Than Acetate in *A. woodii* and *C. autoethanogenum*

Acetyl-CoA acts as a precursor, not only for the production of acetate, but also for the formation of products like ethanol (Köpke et al., 2011a; Basen et al., 2014; Müller, 2014; Lo et al., 2015), butanol (Dürre et al., 1992), lactate (Gladden, 2004), 2,3 butanediol (Syu, 2001; Köpke et al., 2011b; Hess et al., 2015), acetone (Dürre et al., 1992; Hoffmeister et al., 2016), isobutene (van Leeuwen et al., 2012) or isoprene (Diner et al., 2018; Figure 6). As deduced above, the reduction of CO_2_ to acetyl-CoA with H_2_ in *A. woodii* requires 0.7 ATP (Figure 4A), whereas with CO as donor, 0.5 ATP is produced (Figure 4B). *C. authoethanogenum* has an energy demand of 0.6 ATP when reducing CO_2_ to acetyl-CoA with H_2_ (Figure 5C), whereas with CO as donor, the ATP balance is zero (Figure 5D). Therefore, if the further conversion of acetyl-CoA to the desired product gains ATP, the production from CO will be possible. Whereas, from H_2_ + CO_2_ it depends on the amount of ATP produced. In contrast, if the pathway to the desired product from acetyl-CoA requires an input of ATP, the production from H_2_ + CO_2_ will not be possible. With CO as electron and carbon source it depends on the amount of energy required. As mentioned above, the overall ATP yield can be higher in *C. authoethanogenum*, when an electron-bifurcating methylene-THF reductase is assumed. In this case the ATP balance is zero, when CO_2_ is reduced to acetyl-CoA with H_2_ (Figure 5A), whereas in contrast 0.5 ATP per acetyl-CoA is produced with CO as substrate (Figure 5B).

Ethanol can be made from acetyl-CoA by two reduction steps *via* acetaldehyde (Figure 6). The reduction of acetyl-CoA to ethanol with NADH as electron donor is close to equilibrium (ΔG^0′^ = −6.2 kJ/mol) and catalyzed by NADH-dependent enzymes like the bifunctional AdhE (Thauer et al., 1977; Goodlove et al., 1989; Peng et al., 2008; Yakushi and Matsushita, 2010; Extance et al., 2013; Bertsch et al., 2016). Thus, 2 NADH are required for the reduction of acetyl-CoA to ethanol. When a product like ethanol is formed from H_2_ + CO_2_ *via* acetyl-CoA, the ATP gained in the acetate kinase reaction is missing and the overall ATP gain is negative with a demand of 0.7 ATP/per mol of ethanol in *A. woodii*. In addition, two more reducing equivalents are required; the reduction of 2 NAD^+^ with H_2_ as electron donor yields 0.6 ATP by action of hydrogenase, Rnf complex, and ATP synthase. In sum, ethanol formation has an energy demand of 0.1 ATP (Table 1). With CO as electron donor, ethanol formation would be possible with an ATP yield of 1.7 ATP/ethanol (Table 1). The same holds true for *C. authoethanogenum*. It was shown, that *C. autoethanogenum* can produce ethanol when growing on CO (Abrini et al., 1994; Abubackar et al., 2015) or H_2_ + CO_2_ as electron donor (Mock et al., 2015). Ethanol formation from H_2_ + CO_2_ has an energy demand of 0.2 ATP and with CO an ATP gain of 1.4 ATP/ethanol (Table 2). However, in *C. autoethanogenum* ethanol production from H_2_ + CO_2_ could be possible, in comparison to *A. woodii*, if a bifurcating methylene-THF reductase is assumed. In this scenario, *C. autoethanogenum* would gain 0.4 ATP per produced ethanol (Table 2). With CO the energetics for ethanol production is even better with 1.9 ATP/ethanol (Table 2).

**Table 1:** ATP yield for the synthesis of products from acetyl-CoA with H_2_ + CO_2_ or CO as electron donor in *A. woodii*.

**Product**	**Key enzymes/intermediates**	**Conversion (acetyl-CoA as precursor)**	**ATP yield**	
			**H_2_+CO_**2**_**	**CO**
Acetate	Acetate kinase	acetyl-CoA→acetate	0.3	1.5
Ethanol	AlDH/ADH	acetyl-CoA→ethanol	−0.1	1.7
	AOR + ADH		0.3	2.1
Butanol	BDH	2 acetyl-CoA→butanol	−0.2	3.4
	BDH, bifurcating Bcd		0.4	4.0
	AOR		0.2	3.8
	AOR, bifurcating Bcd		0.8	4.4
Isoprene	Mevalonate	3 acetyl-CoA→isoprene + CO_2_	−4.5	−0.3
Lactate	NADH-dependent LDH	acetyl-CoA + CO_2_→lactate	−0.7	1.1
	Bifurcating LDH		−0.1	1.7
2,3-Butanediol	Acetolactate synthase	2 acetyl-CoA→2,3-butanediol	−1.7	1.6
Acetone	Acetoacetate	2 acetyl-CoA→acetone + CO_2_	−0.4	2.0
Isobutene	Acetone, 3-OH-isovalerate	3 acetyl-CoA→isobutene + 2 CO_2_	−2.1	1.5

**Table 2:** ATP yield for the synthesis of products from acetyl-CoA with H_2_ + CO_2_ or CO as electron donor in *C. autoethanogenum*.

**Product**	**Key enzymes/intermediates**	**Conversion (acetyl-CoA as precursor)**	**Methylene-THF reductase**		**ATP yield**	
			**bifurcating**	**not bifurcating**	**H_2_+CO_**2**_**	**CO**
Acetate	Acetate kinase	acetyl-CoA→acetate	–	+	0.4	1.0
Ethanol	AlDH /ADH	acetyl-CoA→ethanol	–	+	−0.2	1.4
	AOR + ADH		–	+	0.4	1.9
Butanol	BDH	2 acetyl-CoA→butanol	–	+	−0.4	2.8
	BDH, bifurcating Bcd		–	+	0.1	3.3
	AOR		–	+	0.1	3.3
	AOR, bifurcating Bcd		–	+	0.6	3.8
Isoprene	Mevalonate	3 acetyl-CoA→isoprene + CO_2_	–	+	−4.7	−0.9
Lactate	NADH-dependent LDH	acetyl-CoA + CO_2_→lactate	–	+	−0.7	0.9
	Bifurcating LDH		–	+	−0.2	1.4
2,3-Butanediol	Acetolactate synthase	2 acetyl-CoA→2,3-butanediol	–	+	−1.8	1.2
Acetone	Acetoacetate	2 acetyl-CoA→acetone + CO_2_	–	+	−0.5	1.7
Isobutene	Acetone, 3-OH-isovalerate	3 acetyl-CoA→isobutene + 2 CO_2_	–	+	−2.2	1.0
Acetate	Acetate kinase	acetyl-CoA→acetate	+	–	1.0	1.5
Ethanol	AlDH /ADH	acetyl-CoA→ethanol	+	–	0.4	1.9
	AOR + ADH		+	–	1.2	2.4
Butanol	BDH	2 acetyl-CoA→butanol	+	–	0.2	3.3
	BDH, bifurcating Bcd		+	–	0.8	3.9
	AOR		+	–	0.6	3.8
	AOR, bifurcating Bcd		+	–	1.1	4.3
Isoprene	Mevalonate	3 acetyl-CoA→isoprene + CO_2_	+	–	−2.3	−0.2
Lactate	NADH–dependent LDH	acetyl-CoA + CO_2_→lactate	+	–	0.2	1.4
	Bifurcating LDH		+	–	0.8	1.9
2,3-Butanediol	Acetolactate synthase	2 acetyl-CoA→2,3-butanediol	+	–	−1.2	1.9
Acetone	Acetoacetate	2 acetyl-CoA→acetone + CO_2_	+	–	0.1	2.0
Isobutene	Acetone, 3-OH-isovalerate	3 acetyl-CoA→isobutene + 2 CO_2_	+	–	−1.6	1.6

However, there is a second way of producing ethanol from acetyl-CoA in acetogens. Aldehyde:ferredoxin oxidoreductases (AOR) are capable of catalyzing the reversible reduction of an acid to the corresponding aldehyde, in this case, the reduction of acetate to acetaldehyde (White et al., 1989; Nissen and Basen, 2019). The importance of AOR enzymes are discussed in more detail later in this review. The redox potential of acetate/acetaldehyde (E_0_' = −580 mV) is so negative that a low potential electron donor such as ferredoxin is required (Thauer et al., 1977). The further reduction of acetaldehyde to ethanol could be catalyzed by a monofunctional alcohol dehydrogenase (ADH) (Goodlove et al., 1989), or by the same AdhE as described above. Indeed, *C. autoethanogenum* has an AOR enzyme that together with ADH converts acetate to ethanol (Mock et al., 2015). Under these conditions, ethanol production from H_2_ + CO_2_ yields 0.4/1.2 ATP/ethanol, while ethanol production from CO gains 1.9/2.4 ATP/ethanol (depending on the MTHFR reaction) (Table 2). *A. woodii* does not have an AOR (Poehlein et al., 2012), but if one is implemented by metabolic engineering, ethanol formation from H_2_ + CO_2_ would be possible with an ATP yield of 0.3 ATP/ethanol (Table 1). With CO as electron donor, the energetics for ethanol production with an implemented AOR in *A. woodii* are even better, yielding 2.1 ATP/ethanol (Table 1).

The production of other valuable products in *A. woodii* or *C. autoethanogenum* such as lactate, butanol, acetone, isobutene or 2,3-butanediol has an energy demand with H_2_ + CO_2_ but a higher net ATP gain with CO as electron donor (Tables 1, 2). To form lactate, acetyl-CoA has to be first carboxylated to pyruvate by a pyruvate:ferredoxin oxidoreductase (Figure 6; Furdui and Ragsdale, 2000). Using NADH as electron donor, pyruvate is further reduced to lactate (ΔG^0′^ = −25 kJ/mol) by a lactate dehydrogenase (LDH) (Gladden, 2004). *A. woodii* has an electron-bifurcating lactate dehydrogenase (bLDH), which increases the amount of ATP produced *via* chemiosmosis (Weghoff et al., 2015). Even if a bifurcating LDH is involved, production of lactate from H_2_ + CO_2_ still requires energy in *A. woodii* or *C. autoethanogenum* by −0.1 or −0.2/−0.7 ATP/lactate (depending on the MTHFR reaction) and, thus lactate production from H_2_ + CO_2_ is not possible (Tables 1, 2). However, if an electron-bifurcating MTHFR is assumed, lactate production with or without a bifurcating LDH from H_2_ + CO_2_ is possible (0.2 or 0.8 ATP/lactate) in *C. autoethanogenum* (Table 2). If CO is used as electron donor, the production of lactate with *A. woodii* or *C. autoethanogenum* will always gain ATP, independent of any electron bifurcation event (MTHFR, bLDH) (Tables 1, 2). Due to the energetics, *A. woodii* can not produce lactate from H_2_ + CO_2_ (Schoelmerich et al., 2018). In contrast, *C. autoethanogenum* has been shown to produce lactate from syngas (Köpke et al., 2011b; Liew et al., 2017).

For the production of butanol, 2 molecules of acetyl-CoA are required (Dürre et al., 1992). Two molecules of acetyl-CoA are condensed to acetoacetyl-CoA which is reduced to 3-hydroxypropionyl-CoA with NADH (Figure 6). After water is split off, crotonyl-CoA is reduced to butyrate by a butyryl-CoA dehydrogenase (Bcd). Due to the relative positive redox potential of the crotonyl-CoA/butyryl-CoA couple (E_0^′^_ = −10 mV), NADH-dependent Bcds can couple crotonyl-CoA reduction to ferredoxin reduction (bBcD) (Li et al., 2008). If the Bcd is not electron bifurcating, the pathway requires 4 NADH and yields 1.2 ATP/butanol for *A. woodii* or 1.6/2.2 ATP/butanol for *C. authoethanogenum* (depending on the MTHFR reaction). If the Bcd is electron bifurcating, the additional conservation of energy *via* the Rnf complex leads to a total ATP yield of 1.8 ATP/butanol for *A. woodii* or 2.6/3.2 ATP/butanol for *C. authoethanogenum* (depending on the MTHFR reaction). Since 1.4 ATP in *A. woodii* or 1.2 ATP in *C. authoethanogenum* have to be invested to supply 2 acetyl-CoA, the butanol production from H_2_ + CO_2_ *via* butyraldehyde dehydrogenase is only possible, if a bifurcating Bcd is involved (Tables 1, 2). In case of *C. authoethanogenum* the energetics for butanol production from H_2_ + CO_2_ without a bifurcating Bcd (0.2 ATP/butanol) only gains ATP, if an electron-bifurcating MTHFR is assumed (Table 2).

Butanol production can also be achieved by reducing butyrate to butyraldehyde (Nissen and Basen, 2019). AORs have been shown to reduce a broad range of acids to the corresponding aldehydes, also the reduction of butyrate to butyraldehyde (Ni et al., 2012; Perez et al., 2013). The requirement of Fd^2−^ reduces the energy yield *via* chemiosmosis, but the formation of butyrate from butyryl-CoA yields 1 ATP *via* substrate-level phosphorylation. Butanol production *via* AORs is energetically possible for *A. woodii* and *C. authoethanogenum* independent on the pathway and electron bifurcation (bBcD, MTHFR) (Tables 1, 2). With CO as electron donor, production of butanol will be, in any case, strongly energy positive, yielding 3.4–4.4 ATP/butanol for *A. woodii* (depending on the pathway) or 2.8–3.8/3.3–4.3 ATP/butanol for *C. authoethanogenum* (depending on the pathway/ MTHFR reaction) (Tables 1, 2).

For the production of 2,3-butanediol, 2 pyruvate are condensed and decarboxylated, yielding acetolactate (Figure 6; Hess et al., 2015). This is further decarboxylated, giving rise to acetoin. The reduction of acetoin with NADH yields 2,3-butanediol. In sum, 2 acetyl-CoA are reduced with 2 Fd^2−^ and 2 NADH to 2,3-butanediol. The use of H_2_ or CO as external electron donor makes big differences in the energy balance: with H_2_, in *A.woodii* 1.7 ATP have to be invested for the synthesis of one 2,3-butanediol, with CO 1.6 ATP are produced for every 2,3-butanediol synthesized (Table 1). Using H_2_ as electron donor in *C. authoethanogenum* 2,3-butanediol production is not possible, even if the MTHFR is electron bifurcating, because the ATP/2,3-butanediol ratio is strongly ATP-dependent with −1.8/−1.2 ATP/2,3-butanediol (depending on the MTHFR reaction) (Table 2). Again, the energetic favorable reduction power of CO enhances the ATP yield also during 2,3-butanediol fermentation (1.2/1.9 ATP/2,3-butanediol; depending on the MTHFR reaction), which makes the production of 2,3-butanediol in *C. authoethanogenum* possible (Köpke et al., 2011b).

Recently, *A. woodii* was shown to produce acetone from H_2_ + CO_2_ after implementation of the corresponding pathway from *Clostridium acetobutylicum*, but only as a side product in small amounts (Hoffmeister et al., 2016). During the production of acetone 2 acetyl-CoA are condensed to acetoacetyl-CoA, which is further converted to acetoacetate and then to acetone (Figure 6; Dürre et al., 1992). The production of acetone from H_2_ + CO_2_ by *A. woodii* would require an input of ATP (-0.4 ATP/acetone), while production from CO would be coupled to ATP production (2.0 ATP/acetone) (Table 1). In comparison, acetone production from H_2_ + CO_2_ by *C. authoethanogenum* is possible, if an electron-bifurcating MTHFR is assumed (Table 2). The overall ATP/acetone yield is in this case positive, with 0.1 ATP per produced acetone (Table 2). With CO, *C. authoethanogenum* has an even better ATP yield with 1.7/2.0 ATP/acetone (depending on the MTHFR reaction) (Table 2).

Acetone can be a precursor for further products. For example, for the production of isobutene (Figure 6; van Leeuwen et al., 2012). With the patented enzyme system for the acetylation of acetone to 3-hydroxy-isovalerate, followed by the ATP-dependent decarboxylation, isobutene could be produced in microorganisms (van Leeuwen et al., 2012). Because an even higher ATP investment is necessary, compared to the production of acetone, the energetics for *A. woodii* or *C. authoethanogenum* with H_2_ + CO_2_ as substrate are much worse (Tables 1, 2). The production of isobutene from H_2_ + CO_2_ by *A. woodii* would require an input of ATP (−2.1 ATP/isobutene), while production from CO would be coupled to ATP production (1.5 ATP/isobutene) (Table 1). Using *C. authoethanogenum* for the production of isobutene, an input of ATP of −2.2/−1.6 per isobutene (depending on the MTHFR reaction) with H_2_ + CO_2_ is needed (Table 2). With CO as electron donor, *C. authoethanogenum* has an ATP gain with 1.0/1.6 ATP/isobutene (depending on the MTHFR reaction) (Table 2).

The production of isoprene is even more energy consuming. To produce isoprene a non-mevalonate pathway has been engineered in acetogens (Diner et al., 2018). The mevalonate pathway starts with the condensation of 2 acetyl-CoA to acetoacetyl-CoA (Figure 6). The addition of a third acetyl-CoA yields 3-hydroxy-3-methyl-glutaryl-CoA (HMG-CoA), which is reduced by a NAD^+^-dependent HMG-CoA reductase to mevalonate. After two consequent ATP-dependent phosphorylations, diphosphomevalonate is decarboxylated at the expense of ATP, giving rise to isopentenyl diphosphate (IPP) (Kuzuyama, 2002). After an isomerization, the diphosphate bond is hydrolyzed, yielding isoprene. Thus, in total, the conversion of 3 acetyl-CoA to isoprene requires 2 NADPH and 3 ATP. Because of the high energy input needed, both *A. woodii* or *C. authoethanogenum* have an ATP-demand per produced isoprene when growing on H_2_ + CO_2_ or CO as substrate under all possible scenarios (Tables 1, 2).

## Overcoming Energetic Barriers

The reduction of CO_2_ with H_2_ or with CO as reductant is energetically limited and currently only two products, ethanol and acetate can be produced with high titers on an industrial scale (Daniell et al., 2012; Bengelsdorf et al., 2018). As outlined above, ethanol formation from H_2_ + CO_2_ *via* acetyl-CoA to acetaldehyde and ethanol requires the net input of ATP and should not be possible with high specificity. However, it is possible in some species, and these species have an enzyme that activates acetate (ΔG^0′^ = −15.4 kJ/mol) with reduced ferredoxin as electron donor, the aldehyde:ferredoxin oxidoreductase (AOR) (White et al., 1989; Chan et al., 1995; Heider et al., 1995; Nissen and Basen, 2019). Therefore, ethanol formation still includes the acetate kinase reaction. Although additional reduced ferredoxin is required as driving force and thus missing as driving force for ATP synthesis, the overall process yields 1.2 ATP/mol ethanol. Acetogens like *A. woodii* that do not have an AOR do not produce ethanol from H_2_ + CO_2_, but with the advent of genetic methods in acetogens, implementing an AOR into the acetogenic metabolism would be the first choice to increase the energetics and overcome the energetic barrier to acetyl-CoA formation. In contrast, *C. autoethanogenum* produces ethanol from H_2_ + CO_2_ (Mock et al., 2015; Liew et al., 2017). The importance of AOR enzymes for higher ethanol production rates were shown, for example, in *Pyrococcus furiosus* or *C. authoethanogenum* (Basen et al., 2014; Liew et al., 2017). An engineered *P. furiosus* strain converted glucose to ethanol *via* acetate and acetaldehyde, catalyzed by the host-encoded aldehyde ferredoxin oxidoreductase (AOR) and heterologously expressed AdhA, in an energy-conserving, redox-balanced pathway (Basen et al., 2014; Müller, 2014). Implementing an indirect ethanol pathway by deletion of *AdhE* in the genome of *C. authoethanogenum* shifted the conversion of acetate to acetaldehyde *via* AOR (Liew et al., 2017). Using this strategy the generated strains produced up to 180% more ethanol and also accumulated up to 38% less of acetate. The first heterologous AOR production in an acetogenic bacterium was recently described for *Clostidium carboxidivorans* (Cheng et al., 2019). A plasmid-based implementation of the AOR genes lead to higher ethanol production rates during growth on glucose. Furthermore strains overexpressing AOR showed CO_2_ re-assimilation during heterotrophic growth on glucose (Cheng et al., 2019). However, the selectivity of AORs to reduce short chain fatty acids, like acetate, can also easily be achieved with simple changes in growth conditions (Abubackar et al., 2015; Mock et al., 2015; Martin et al., 2016; Richter et al., 2016a,b). For syngas-fermenting bacteria, that are using the AOR-ADH pathway, solventogenesis (alcohol production) occurs preferably when growth is limited due to nutrient limitations or at low pH (Daniell et al., 2012; Abubackar et al., 2015; Mock et al., 2015; Al-Shorgani et al., 2018). For example, results from bioreactor studies with continuous CO supply revealed that the shift from high to low pH values improves ethanol production by *C. autoethanogenum* without any accumulation of acetic acid (Abubackar et al., 2015). Low pH is therefore not only beneficial for ethanol production *via* syngas fermentation, but also an effective method for decreasing acetate concentration in the fermentation broth (Abubackar et al., 2015; Martin et al., 2016; Richter et al., 2016a).

The same principle would also apply to other alcohols. Butanol is an interesting biofuel and can be produced by acetogens from the condensation of two mol acetyl-CoA to acetoacetyl-CoA followed by a reduction to butyryl-CoA*, via* a series of reactions known from fatty acid oxidation (see above) (Dürre, 2007; Köpke et al., 2011c). Butyryl-CoA can be either oxidized to butyrate giving one ATP or reduced to butanol without the production of an ATP. In this pathway, the acetate kinase-produced ATP's (2 molecules) are missing and although one ATP is gained by the butyrate kinase, the overall ATP gain is negative and butyrate cannot be produced from H_2_ + CO_2_. With CO as substrate the energetics are a bit better. The oxidation of CO is coupled to more ferredoxin reduction, which can be used as driving force to produce additional ATP. But butanol is not produced for energetic reasons, since a redirection of butyryl-CoA to butanol results in a loss of another ATP. Here, implementation of an AOR by genetic engineering would bring the energetics over the hurdle to produce butanol (Ni et al., 2012; Perez et al., 2013). AORs so far characterized are rather unspecific and can oxidize chain aldehydes (e.g., formaldehyde, acetaldehyde, crotonaldehyde), branched chain aldehydes (e.g., isovalerylaldehyde) as well as aromatic aldehydes (phenylacetaldehyde, benzaldehyde) or reduce acetate and butyrate (Nissen and Basen, 2019). The specificity and efficiency of the AOR could be improved by directed evolution (Turner, 2003; Dalby, 2011).

**Figure 7:**
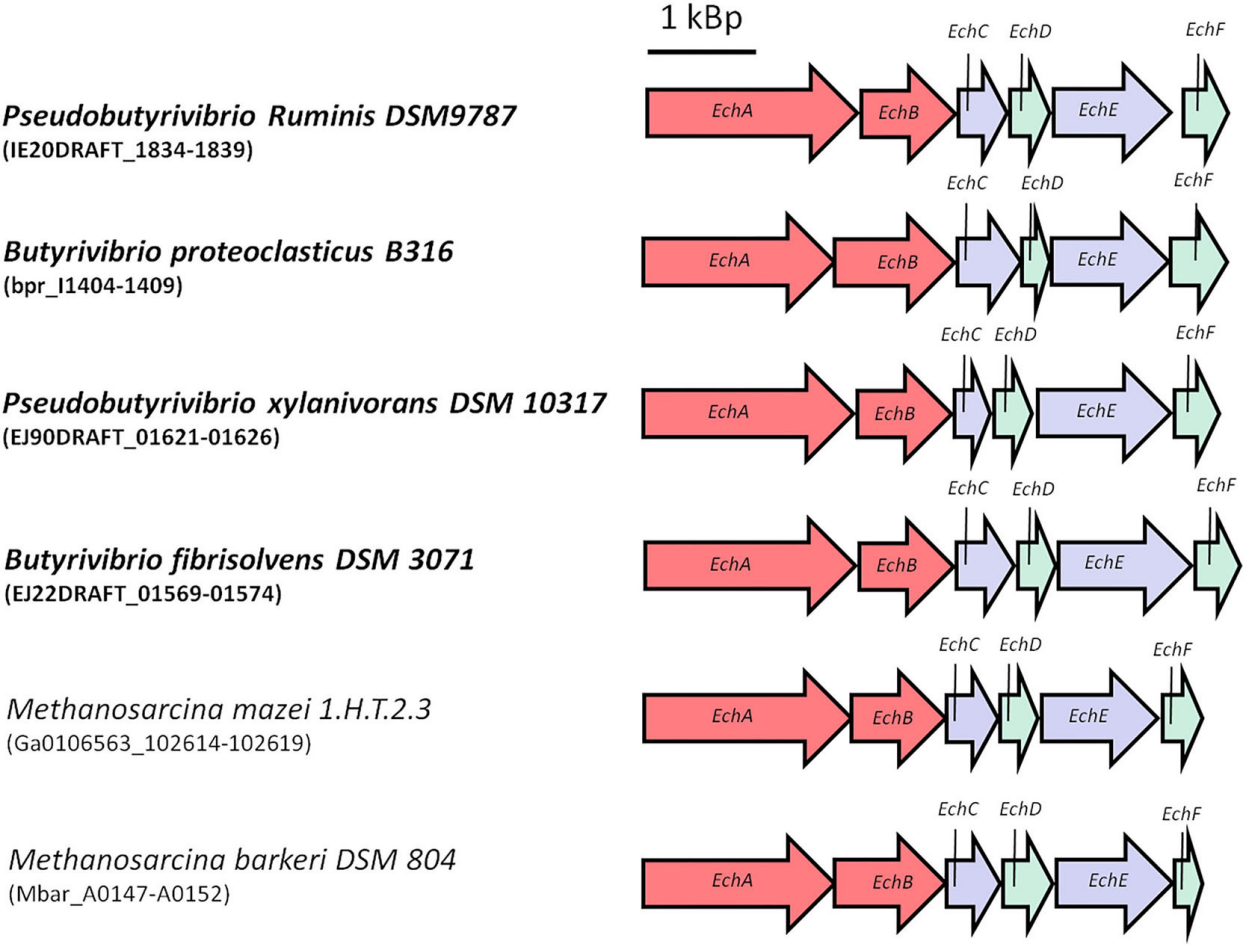
Ech gene-cluster of representative mesophilic anaerobes. Ech genes are highly distributed in the mesophilic *Butyrivibrio* clade. Typical anaerobes are for example *Pseudobutyrivibrio ruminis, Butyrivibrio proteoclasticus, Pseudobutyrivibrio xylanivorans* or *Butyrivibrio fibrisolvens*. Here shown are the Ech gene-cluster-comparison against representative archea *M. mazei* and *M. barkeri*. Ech gene-cluster of mesophilic anaerobes show similar genetic organization.

As outlined above, energy conservation during acetogenesis from H_2_ + CO_2_ involves substrate level phosphorylation as well as electron transport phosphorylation. The former yields one ATP per acetate, the latter much less, in the order of 0.3 and less per mol of acetate. So far, every acetogen examined has only one of the two respiratory enzymes, Ech (Schoelmerich and Müller, 2019) or Rnf (Biegel et al., 2011). Interestingly, Ech complexes have thus far only been found in thermophilic species (Schoelmerich and Müller, 2019). One idea to improve the energetics is to implement by genetic engineering an Ech complex into *A. woodii, C. autoethanogenum* or others, but this requires an Ech complex from a mesophilic bacterium. Fortunately, Ech-containing mesophilic anaerobes do exist, for example in the *Butyrivrio* clade (Figure 7; Hackmann and Firkins, 2015; Schoelmerich et al., 2020). Currently, it is tested whether these can be functionally produced in *A. woodii*. Another obstacle is that *A. woodii* uses Na^+^ as coupling ion for the ATP synthase and the Rnf complex (Hess et al., 2013b; Matthies et al., 2014); ideally, the Ech complex implemented should also translocate Na^+^ but so far, the ion specificity of these Ech complexes as well as others is not known, simply due to the fact that they have never been purified and reconstituted into liposomes. However, even if an Ech complex is a proton and not a sodium ion pump, the proton gradient established would be converted to a Na^+^ gradient by a Na^+^/H^+^ antiporter present in *A. woodii* (Biegel and Müller, 2011). The same principle could be used *vice versa*, but this would require a thermophilic Rnf complex, for example from *Thermotoga maritima* (Kuhns et al., 2020).

Membrane-bound cytochromes are well-known electron carriers in membrane-bound electron transport chains; their presence would immediately suggest an electron transport chain and electron transport phosphorylation in the organism they have been discovered in (Gottwald et al., 1975; Das et al., 1989; Das and Ljungdahl, 2003). Methanogenic archaea also use the Wood-Ljungdahl pathway for CO_2_ fixation and methane formation but how this is coupled to energy conservation has been hotly debated a while ago (Blaut and Gottschalk, 1984; Lancaster, 1989). Some favored substrate level phosphorylation by an unknown reaction in the WLP, other some sort of to be discovered electron transport phosphorylation. The discovery of b559-type cytochromes in *M. barkeri* in 1979 by Kühn and Gottschalk was a ground-breaking study that excited the entire field and paved the road to the discovery of electron transport phosphorylation in this model methanogen (Kühn et al., 1979; Blaut and Gottschalk, 1984). Later, methanophenazine (Abken et al., 1998) was found as additional electron carrier in these cells and the heterodisulfide reductase as electron acceptor (Bäumer et al., 1998). Now, different electron transport chains with different electron input modules in different methanogens are well-established (Deppenmeier, 2002; Schlegel and Müller, 2013; Welte and Deppenmeier, 2014). In this historic context was the discovery of *b*-type cytochromes in *M. thermoacetica* in 1975 by Gottwald et al. That was immediately taken as indication for cytochrome-dependent electron transport chain as part of a chemiosmotic mechanism of ATP synthesis. Later, reduction of cytochromes as well as the concomitant generation of a membrane potential in vesicles of *C. thermoautotrophicum* was detected (Hugenholtz and Ljungdahl, 1989). The most conclusive evidence that the cytochromes are involved in coupling the WLP to energy conservation was presented by Kamlage and Blaut (1993). By showing that a cytochrome-deficient mutant was no longer able to oxidize methyl groups to CO_2_ or reduce CO_2_ to the level of a methyl group, they laid the foundation that cytochromes are involved as electron carriers in the methylene-THF reductase, a reaction that was already postulated in 1977 by Thauer et al. (1977) to be the most likely energy conserving site since reduction of methylene-THF to Methyl-THF (ΔG^0′^ = −23.1 kJ/mol) with NAD^+^ as reductant is the most exergonic of the pathway. An involvement of cytochromes in CO_2_ reduction is also supported by the finding that nitrate, an alternative electron acceptor in *M. thermoacetica*, represses the synthesis of the *b*-type cytochrome (Fröstl et al., 1996; Arendsen et al., 1999). An involvement of cytochromes in the methylene-THF reductase reaction was also very recently speculated by Keller et al. (2019) for *Thermacetogenium phaeum*. However, it is far from being settled whether or not there is indeed a third, cytochrome-dependent respiratory chain in addition to Ech- and Rnf-containing respiratory chains in acetogens. This must be addressed in future studies using the genetic tools that have been developed. However, it should be noted that Rnf- and Ech-acetogens such as *A. woodii* and *T. kivui* do not have cytochromes (Müller, 2003). Others seem to have Rnf plus cytochromes such as *Sporomusa* (Kamlage et al., 1993; Poehlein et al., 2013) or *Clostridium aceticum* (Poehlein et al., 2015) or Ech plus cytochromes such as *M. thermoacetica* (Gottwald et al., 1975; Pierce et al., 2008). Important for the context here is, that the current industrially relevant cells do not have cytochromes. However, implementing cytochromes by genetic engineering is not an easy task since a rather extensive biogenesis machinery as well as genes encoding biosynthesis of quinones are required (Thöny-Meyer, 1997).

In addition to CO_2_, some acetogens can reduce a number of alternative substrates such as pyruvate (Misoph and Drake, 1996), fumarate (Dorn et al., 1978; Matthies et al., 1993), aromatic acrylates (Bache and Pfennig, 1981; Tschech and Pfennig, 1984; Misoph et al., 1996), inorganic sulfur compounds (Beaty and Ljungdahl, 1990, 1991; Hattori et al., 2000), and nitrate or nitrite (Seifritz et al., 1993, 2003; Fröstl et al., 1996; Arendsen et al., 1999). For industrial applications, the electron acceptor would have to be added as well as the product removed from the fermenter, thus raising the costs of operation and the price of the product. Of these alternative electron acceptors, the use of aromatic acrylates, nitrate and nitrite have been studied to some extent (Bache and Pfennig, 1981; Tschech and Pfennig, 1984; Seifritz et al., 1993, 2003; Fröstl et al., 1996; Arendsen et al., 1999). Aromatic acrylates such as caffeate are reduced by *A. woodii* simultaneously with CO_2_ when H_2_ or fructose is the electron donor, or sequential with CO_2_ first, when methanol is the electron donor (Dilling et al., 2007). Caffeate respiration is linked to energy conservation *via* Rnf and ATP synthase and co-utilization of caffeate and CO_2_ would enhance the ATP level (Imkamp and Müller, 2002; Hess et al., 2013a). However, caffeate is rather toxic and cells do not tolerate more than ~ 5 mM (Parke and Ornston, 2004), which, in addition to the costs, makes the approach economically less favorable. Nitrate is rather inexpensive but the effect of nitrate is discussed very controversially. Under heterotrophic conditions, nitrate is stimulatory but lithotrophic growth of *M. thermoacetica* is inhibited (Seifritz et al., 2002). The basis for this inhibition remains to be elucidated, one study argues that the activity of enzymes of the WLP is not altered but only cytochromes disappear (Fröstl et al., 1996) whereas the other argues that also the activities of key enzymes are down regulated by nitrate (Arendsen et al., 1999). Mechanistically, it is not known whether nitrate reduction is coupled to energy conservation *via* a nitrate-dependent ion-motive respiratory chain or whether the presence of nitrate redirects electrons to the Rnf- and Ech-complexes thus providing more fuel for chemiosmotic ATP synthesis. In contrast, *C. ljungdahlii* co-utilized CO_2_ and nitrate and this enhanced biomass formation from H_2_ + CO_2_ (Emerson et al., 2019). Unfortunately, ethanol production from H_2_ + CO_2_ was strongly inhibited by nitrate, indicating that the electron from acetyl-CoA reduction to ethanol goes to nitrate. However, in pH-controlled bioreactors nitrate improved growth and ethanol formation but resulted in stochastic inhibition events (Klask et al., 2020). Clearly, more basic research has to be done on nitrate reduction and its possible coupling to energy conservation and in addition, one has to remember that nitrate reduction is only observed in a few species and, for example, not in *A. woodii*.

So far, we have discussed alterations in the pathway of carbon and electrons in acetogenesis from H_2_ + CO_2_ or CO to increase chemiosmotic ATP synthesis. An alternative is to bring in a reaction (sequence) that is coupled to substrate level phosphorylation. To phosphorylate ADP the reaction must have a phosphoryl group transfer potential that is more negative than −31.8 kJ/mol (Thauer et al., 1977). This restricts the reactions to only a few:

Creatine kinase:

(3) $$\begin{array}{l} \text{creatinephosphate}+{\text{H}}_{2}\text{O}\to \text{creatine}+{\text{P}}_{\text{i}}\\ \;(\Delta {\text{G}}^{0^{\text{′}}}=-43.3\text{ kJ}/\text{mol}) \end{array}$$

Arginine kinase:

(4) $$\begin{array}{l} \text{argininephosphate}+{\text{H}}_{2}\text{O}\to \text{arginine}+{\text{P}}_{\text{i}}\\ \;(\Delta {\text{G}}^{0^{\text{′}}}=-45.0\text{ kJ}/\text{mol}) \end{array}$$

Carbamate kinase:

(5) $$\begin{array}{l} \text{carbamylphosphate}+{\text{H}}_{2}\text{O}\to \text{carbamate}+{\text{P}}_{\text{i}}\\ \;(\Delta {\text{G}}^{0^{\text{′}}}=-39.3\text{ kJ}/\text{mol}) \end{array}$$

Succinyl-CoA synthetase:

(6) $$\begin{array}{l} \text{succinyl}-\text{CoA}\to succinate+\text{HS}-\text{CoA}\\ \;(\Delta {\text{G}}^{0^{\text{′}}}=-27.0\text{ kJ}/\text{mol}) \end{array}$$

Acetate kinase:

(7) $$\begin{array}{l} \text{acetylphosphate}+{\text{H}}_{2}\text{O}\to \text{acetate}+{\text{P}}_{\text{i}}\\ \;(\Delta {\text{G}}^{0^{\text{′}}}=-44.8\text{ kJ}/\text{mol}) \end{array}$$

Butyrate kinase:

(8) $$\begin{array}{l} \text{butyrylphosphate}+{\text{H}}_{2}\text{O}\to \text{butyrate}+{\text{P}}_{\text{i}}\\ \;(\Delta {\text{G}}^{0^{\text{′}}}=-35.6\text{ kJ}/\text{mol}) \end{array}$$

Phosphoglycerate kinase:

(9) $$\begin{array}{l} 1,3-\text{bisphosphoglycerate}+{\text{H}}_{2}\text{O}\to 3-\text{phosphoglycerate}\\ \;+{\text{P}}_{\text{i}}\\ \;(\Delta {\text{G}}^{0^{\text{′}}}=-51.9\text{ kJ}/\text{mol}) \end{array}$$

Pyruvate kinase:

(10) $$\begin{array}{l} \text{phosphoenolpyruvate}+{\text{H}}_{2}\text{O}\to \text{pyruvate}+{\text{P}}_{\text{i}}\\ \;(\Delta {\text{G}}^{0^{\text{′}}}=-51.6\text{ kJ}/\text{mol}) \end{array}$$

Arginine kinase and creatine kinase (equation 3 and 4) play an essential role in ATP buffering systems in invertebrates (Wallimann et al., 1992) and are not useful here since they require previous activation of the substrate by ATP. Reaction 4 is part of the arginine deaminase pathway, a pathway found in eukaryotes (Novak et al., 2016) as well as prokaryotes (Deibel, 1964) that provide ATP from a fermentative pathway. In that pathway arginine is converted to ornithine, ammonium, and CO_2_, while generating ATP from ADP and phosphate. The enzymes involved in the three steps of the pathway are arginine deiminase (ADI) (Petrack et al., 1957), ornithine transcarbamylase (OTC) (Kalman et al., 1965; Hernandez and Johnson, 1967), and carbamate kinase (CK) (Grisolia et al., 1962). The first reaction, catalyzed by ADI, is the deamination of arginine to yield citrulline and ${\text{NH}}_{4}^{+}$ (Petrack et al., 1957). OTC then catalyzes the conversion of citrulline and inorganic phosphate into carbamoyl-phosphate and ornithine (Kalman et al., 1965; Hernandez and Johnson, 1967). Finally, CK catalyzes the hydrolysis of carbamoyl phosphate to form CO_2_ and ${\text{NH}}_{4}^{+}$, while the phosphate group is used to regenerate ATP from ADP (Grisolia et al., 1962). Indeed, feeding arginine (20 mM) promoted growth advantages in *A. woodii* (Beck et al., 2020). A 69% higher maximal OD_600_ and about 60% lower acetate yield per biomass was obtained in the presence of arginine (Beck et al., 2020). However, the costs of arginine and the removal of ornithine and ammonium have to be considered. Succinly-CoA synthetase is a reaction of the tricarbonic acid cycle (TCC) (Hager, 1962); this could be useful by feeding citrate which is converted by the enzymes of the TCC to succinate. Uptake of citrate (Dimroth and Thomer, 1986) and export of succinate (Kimmich et al., 1991) must be ensured by the appropriate transporter; another disadvantage is that the reaction sequence requires four electrons (2 NADH). Acetate kinase and butyrate kinase have been discussed above as part of the butyrate/butanol pathway. Intermediates of this pathway are CoA esters, which are expensive, instable and probably not taken up (Banis et al., 1976). Feeding the precursors acetate or acetoacetate would require an ATP-dependent activation, so that the net ATP gain by SLP would be zero.

An additional strategy is to provide organic electron donors that are acetogenic substrates. It is known for some acetogens that they can utilize different substrates simultaneously, for example H_2_ + CO_2_ together with glucose (Braun and Gottschalk, 1981). The WLP is ideally suited for capturing CO_2_ to increase the product yields. Homoacetogenesis according to equation 11 and 12 can be regarded as mixotrophic growth: sugar is oxidized to acetate giving rise to ATP synthesis by the reactions of pyruvate kinase, phosphoglycerate kinase and acetate kinase.

(11) $$\begin{array}{l} \text{glucose}+4\text{ ADP}+4{\text{ P}}_{\text{i}}\to 2\text{ acetate}+2\text{ C}{\text{O}}_{2}\\ \;+4\text{ ATP}+2{\text{ H}}_{2}\text{O}+8\;\left[\text{H}\right] \end{array}$$

(12) $$\begin{array}{l} 8\;\left[\text{H}\right]+2\text{ C}{\text{O}}_{2}+x\;ADP+x\;P_{i}\to acetate\\ \;+2\;H_{2}O+x\;ATP \end{array}$$

The electrons gained during glycolysis are captured by CO_2_ reduction (Schuchmann and Müller, 2016). Alternatively, interspecies H_2_ transfer can be used instead of the WLP to get rid of surplus reducing equivalents as shown for *A. woodii* (Winter and Wolfe, 1980; Wiechmann et al., 2020) or *T. kivui* (Moon et al., 2020). In principle any oxidative pathway can be hooked up to the WLP; this is what makes the WLP and acetogens so interesting for biotechnology. On the downside, mixotrophy is limited by the redox balance (Schuchmann and Müller, 2016). Reduction of 2 CO_2_ to acetate requires 8 electrons. Therefore, the first requirement for a pathway coupled to the WLP is the release of 8 electrons per 2 CO_2_ produced to allow complete CO_2_ fixation. This is the case for oxidation of glucose to 2 acetate by typical fermentations (Schuchmann and Müller, 2016). However, this approach is limited to end products with the same oxidation state as acetate, since glucose oxidation does not provide enough electrons. Electron limitation can be overcome by supplying additional electron donors such as H_2_ or electrons provided by a cathode for direct electron uptake (Bajracharya et al., 2015; Philips, 2020). In this case, H_2_ provides an unlimited source of reducing power (Liu and Suflita, 1995). Under these conditions, ethanol production from glucose and H_2_ according to equation 13 is possible.

(13) $$\begin{array}{l} \text{glucose}+6{\text{ H}}_{2}\to 3\;ethanol+3\;H_{2}O\\ \;(\Delta {\text{G}}^{0^{\text{′}}}=-337\text{ kJ}/\text{mol}) \end{array}$$

Other examples of acetogenic substrates are primary (Eichler and Schink, 1984, 1985) and secondary alcohols (Zellner and Winter, 1987). Oxidation of ethanol under anaerobic conditions is only possible by specialized bacteria or archaea that either transfer the electrons to H_2_ which is then used by a syntrophic partner or to an electron acceptor such as CO_2_ or sulfate (Eichler and Schink, 1985; Seitz et al., 1990; Schink and Friedrich, 1994; Schink, 1997). Butandiol oxidation requires the presence of CO_2_ according to equation 14.

(14) $$\begin{array}{l} 42,3-\text{butanediol}+6\text{ C}{\text{O}}_{2}+5.3\text{ ADP}+5.3{\text{ P}}_{\text{i}}\to 11\text{ acetate}\\ \;+5.3\text{ ATP}+3.3{\text{ H}}_{2}\text{O} \end{array}$$

The energy released in this reaction (calculated without phosphorylation of ADP) is−89 kJ/mol 2,3-butanediol. The same is true for oxidation of methanol (equation 15) or ethanol (equation 16).

(15) $$\begin{array}{l} 4\text{ methanol}+2\text{ C}{\text{O}}_{2}+2.5\text{ ADP}+2.5{\text{ P}}_{\text{i}}\to 3\text{ acetate}\\ \;+2{\text{ H}}_{2}\text{O}+2.5\text{ ATP} \end{array}$$

(16) $$\begin{array}{l} 2\text{ ethanol}+2\text{ C}{\text{O}}_{2}\to 3\text{ acetate} \end{array}$$

The strategies described so far all apply to pure cultures of acetogens. A fundamentally different strategy is to use consortia of different organisms (Stams, 1994; Stams and Plugge, 2009; Morris et al., 2013; Kouzuma et al., 2015; Angenent et al., 2016). The main products of acetogens such as acetate and ethanol are the substrates for chain elongation *via* reverse β-oxidation (Grootscholten et al., 2013; Angenent et al., 2016). Through a combination of syngas fermentation and chain elongation, C_4_, C_6_ and C_8_ products have been obtained by mixed cultures in open culture fermentation (Angenent et al., 2016). Alternatively, the products of H_2_ + CO_2_ or syngas fermentation, ethanol and/or acetate, can be fed to pure cultures of anaerobes such *Clostridium kluyveri* (Barker and Taha, 1942) to produce n-caproic acid (Barker and Taha, 1942; Gildemyn et al., 2017) or to aerobes such as yeasts (Kerbs et al., 1952; Okada et al., 1981) or *Acinetobacter baylyi* (Salcedo-Vite et al., 2019). Using this approach, the entire tool box is available for the production of higher value compounds.

## Conlcuding Remarks

In times of global climate change alternatives must be explored for a future-proof (bio)technology and energy industry. Renewable energy sources must be used in processes that do not emit CO_2_ or, even better, capture CO_2_ and convert it into valuable compounds that are otherwise made petrochemically. In addition, a future-proof energy industry must take into account molecular H_2_ as promising energy carrier. Different biological solutions are available that solve the most urgent future problems of humanity and are currently tested. Among those are the acetogenic bacteria with their outstanding options to solve these problems. They are the most efficient biocatalysts for H_2_ storage around and their activity is orders of magnitude higher than any chemical catalyst. They are already used on an industrial scale to convert syngas into ethanol and many more products are currently under investigation. They can not only use C_1_ gases but also methanol, other primary and secondary alcohols, carbonic acids as well as sugars as driving force for CO_2_ reduction and there is no other group of anaerobes that has nearly the same metabolic flexibility for CO_2_ reduction than acetogens. The construction of new acetogenic bioplatforms is still in its infancy but steadily increasing; the genetic tool box required is also steadily increasing. Acetogens have a CO_2_ fixation pathway that is energy neutral and does not need additional ATP when acetate is the product. Other products that originate from acetyl-CoA require net input of ATP and, therefore, the main obstacle to overcome in acetogenic fermentations is the energy barrier. This review summarized current knowledge on the strategies to increase the energy charge of the bacteria, some of which have been tested; others could be tested in the future. The road is paved for acetogens to become the key players to achieve a better and more sustainable future for all.

## Author Contributions

VM conceived and designed the review article. AK and VM wrote the manuscript and designed the figures.

## Conflict of Interest

The authors declare that the research was conducted in the absence of any commercial or financial relationships that could be construed as a potential conflict of interest.
